# Exploratory graph analysis on the Connor–Davidson Resilience Scale (CD-RISC) among older adults in China

**DOI:** 10.1038/s41598-023-46854-x

**Published:** 2023-11-15

**Authors:** Yujie Wang, Jixiang Xu, Shitong Yang, Junjia Jiang, Junling Gao

**Affiliations:** 1 School of Public Health, Fudan University, Shanghai, China; 2 Collaborative Innovation Cooperative Unit of National Clinical Research Center for Geriatric Diseases, Shanghai, China; 3 Core Unit of Shanghai Clinical Research Center for Geriatric Diseases, Shanghai, China

**Keywords:** Human behaviour, Risk factors

## Abstract

It is important for healthy aging to understand resilience in depth. This study aims to examine the dimensional structure underlying the Connor–Davidson Resilience Scale (CD-RISC) among Chinese older adults. Exploratory Graph Analysis (EGA) was used to evaluate the dimensional structure of CD-RISC in two large samples: training sample (n = 11,493) and cross-validation sample (n = 7662). Then, Confirmatory Factor Analysis (CFA) was used to compare the fit of the theoretical dimensions with the EGA dimensions. Finially, Generalized Linear Model was used to examine the association between resilience scores and self-rated health (SRH) after controlling other covariates in order to evaluate the predictive value of the EGA dimensions. The EGA indicated two demensions(named foresight and self-adjustment) of the 25-item CD-RISC. The CFA comparison found that the two-demension structure of CD-RISC fit significantly better than the theoretical three-demension structure. After controlling for sociodemographic characteristics, generalized linear model showed that the EGA dimensions has better protective value with SRH. Compared with older adults with lowest quartile of foresight, those with second (odds ratio, OR = 0.68, 95% CI = 0.62 ~ 0.75), third (OR = 0.50, 95% CI = 0.45 ~ 0.56) and fourth quartile (OR = 0.42, 95% CI  =  0.37 ~ 0.48) of foresight had lower odds ratio of poor SRH. Similarly, older adults with the second (OR = 1.11, 95% CI = 1.01 ~ 1.23) and fourth (OR = 0.79, 95% CI = 0.69 ~ 0.90) quartile of self-adjustment also had lower OR of poor SRH than those with lowest quartile of self-adjustment. These findings show that EGA outperforms the traditional methods, which may be helpful to understand resilience deeply. CD-RISC should be interpreted into two aspects among community-dwelling older adults in China, highlighting the significance of the practical value and cultural context of resilience.

In the past decade, research on resilience has drawn attention from a growing number of experts in psychology, psychopathology, sociology, biology, and cognitive neuroscience^1^. American Psychological Association defined resilience as the process of adapting well and even growing in the face of adversity, stress, or trauma^2^. Research has shown that resilience functions as a buffer when people encountering with adversity or stressors, thus improving the ability to adapt to the environment and the efforts to exert control in front of obstacles^3^. During aging process, older adults may more frequently encounter with adversity or stressors including decline of cognitive and physical ability, retirement, death of a loved one, or changes of social network^4^. A better understanding of resilience across the life-course and how it manifests has arisen^5^. According to the healthy aging framework raised by World Health Organization (WHO), a higher level of resilience indicates better intrinsic capacity among older adults thus permiting them to apapt to environment and boost their functional capacity^6^. Previous studies indicated the negative association between resilience and adverse health outcomes, such as hospitalization^7,8^, frailty^9,10^, depression^11,12^, and chronic diseases^13–15^.

Although aging research increasingly incorporates resilience, there is a considerable heterogeneity in the measurement of resilience and its scale structure^16^, which captures different aspects of resilience. Such as, the Brief Resilience Scale (BRS) directly measures one's ability to bounce back or be resilient^17^, and the Resilience Scale for Adults (RSA) contains more intrapersonal protective factors^18^. CD-RISC focuses on resources that can help individuals to recover from and adapt to disruptions or stressful events. Previous research using CD-RISC-25 as a measurement found that in Chinese population, resilience was associated with quality of life^19–22^, depression^23–25^, frailty^10,26^, physical activity and sedentary behaviour^27^. CD-RISC-25 scale was prvoved to have sound psychometric properties among different populations, i.e., adolescents^28^, soldiers^29^, cancer^30^ and depressive patients^31^. However, the CD-RISC is not a stable multidimensional instrument for measuring resilience across the cultures and contexts of countries^32,33^ and lacked of robust replication^34^. The Chinese version of CD-RISC provide theoretically-based and psychometrically sound assessments of strength, optimism and tenacity^34^. Despite the preliminary promising findings, several methodological issues suggest that a reanalysis is warranted^35^. Firstly, cultural differences in item interpretation as well as differences in test settings and analytic strategy may have played a part in the varying factor structures found to date^36^. Different structures of CD-RISC were demonstrated due to the cultural context: a five-factor(25-item) and a one-factor (10-item) solution using a U.S. sample^35,37^, a three-factor solution using a Chinese sample^34^, and a four-factor solution using an Indian sample^38^. Also, items of CD-RISC are highly correlated, and the factors that emerged were in several cases difficult to interpret because they contained items with disparate themes^35^.

Research using psychometric-driven (administration of established questionnaires aimed at quantifying resilience) and data-driven method (use statistical procedures to examine and/or operationalise resilience) are raised to operationalize resilience. However, none of the psychometric evaluations of resilience scales in older adults conducted to date are properly comprehensive, for example: there is no consensus as to the dimensionality of these scales’ latent structure^39^. Compared with the former, one of the data-driven methods, network analysis is advantageous in its capacity to accommodate a range of variable types and investigate the associations between behaviors and symptoms instead of constructs or domains^40^, which means network analysis may show more specific implications of a concept and explore the association with other related aspects. In 2017, Golino developed an innovative approach in exploratory factor analysis titled Exploratory Graph Analysis (EGA) and compared it with other traditional methods. From simulation of thousands of data sets, researchers found EGA outperforms traditional factor analytical and/or eigenvalue-based methods when there exist 2 factors of the scale and factors are highly related^41,42^. With a graphical display, EGA is robust especially in identifying the correct dimensionality when evaluating the instruments with multiple strongly correlated factors (as is the case of CD-RISC) or in large samples^42–44^.

The emphasis of the present study is to verify the original dimensional structure of the CD-RISC-25 among a large sample of community-dwelling Chinese older adults and advance the knowledge regarding the factor structure underlying the CD-RISC structure by conducting a cutting-edge psychometric technique. Specifically, the objectives of this study are to (a) establish the factor structure of the CD-RISC with EGA, and (b) compare the results of the EGA method with its original structure both in Confirmatory Factor Analysis (CFA) and generalized linear models (GLM).

## Methods

### Participants

This cross-sectional study was conducted among community-dwelling Chinese older adults during June 2020 and July 2022 . Sampling method and exclusion criterion has been reported in our previous published research article^10^. 19,970 people were recruited from five cities in China: Shanghai (east of China), Zhuhai(south of China), Panzhihua(west of China), Ordos(north of China) and Hangzhou(east of China). After excluding participants with incomplete questionnaires, a total of 19,155 participants were included. Data was collected from face-to-face surveys using a self-administered questionnaire by trained interviewers. The written informed consent was obtained from all participants and study protocol was approved by the Ethics Committee for Medical Research at the School of Public Health, Fudan University (IRB00002408 & FWA00002399).

### Measures

#### Demographic characteristic

We measured demographic variables of age, gender, marital status and education level using a self-reported demographic checklist. Additionally, we measured the self-rated health (SRH) by asking the participant: "Recently, how do you feel about your health generally?", and the answer is a 5-point Likert response, from 1 (very bad) to 5 (very good). From this item, we merged "very bad" and "bad" into one category for very few participants (141) rating health as "very bad". We created a dichotomous outcome measure of SRH (1 = general or poor; 0 = excellent, very good, or good) for comparing the predictive value in 2 structures.

#### Connor–Davidson resilience scale

The Chinese version of the CD-RISC^34^ was used in the current study, which measures 25 items of three dimensions of resilience: tenacity, strength and optimism. Tenacity refers to equanimity, promptness, perseverance, and sense of control when facing situations of hardship and challenge, and items are "When things look hopeless, I don't give up" and "Under pressure, I focus and think clearly" etc. Strength focuses on the individual's capacity of recovering and becoming strong after a setback and past experiences (e.g. "Past success gives confidence for new challenge", "Things happen for a reason"). Optimism reflects the individual's tendency to look on the positive sides of things and trust in one's personal and social resources(e.g. "I have close and secure relationships", "Sometimes fate or God can help"). Participants were asked to respond to each item on a 5-point Likert scale, from 0 (not true at all) to 4 (true all the time). The higher the score, the higher the level of psychological resilience.

### Statistical analysis

We firstly adopted exploratory factor analysis (EFA) on the total sample and deleted items with less than 0.3 item discrimination or standardized loading matrix < 0.4 if any^45,46^. Despite the large sample size, here we used a novel method to explore the scale structure therefore remained training set as 70% of total participants to train the statistical model. According to 70:30, the total sample was randomly divided into two samples: the scale development sample (sample 1), consisting of 11,493 participants (52.4% female, M_*age*_ = 74.9, SD_*age*_ = 6.3) and the validation sample (sample 2), consisting of 7662 participants (52.3% female, M_*age*_ = 74.9, SD_*age*_ = 6.3). Descriptive statistics were obtained for both the total and the divided sample. We applied EGA in the sample 1 and sample 2 separately. Research has found *bootEGA* is a robust approach for identifying the stability and robustness of dimensionality in multivariate data which allows for the consistency of dimensions and items to be evaluated across bootstrapped EGA results, providing information about whether the data are consistently organized in coherent dimensions or fluctuate between dimensional configurations^47^. Here we use *bootEGA* to assess the stability of the EGA dimensionality estimates and item factor assignments across 1000 bootstrap samples. Third, EFA was adopted in sample 1. Finally, confirmatory factor analysis (CFA) was then adopted in sample 2 to compare the indices of both EGA and EFA dimensionalities. Good fit was determined by values of a comparative fit index (CFI) ≥ 0.95, standardized root mean residual (SRMR) ≤ 0.08, Tucker-Lewis index > 0.90 and root mean square error of approximation (RMSEA) ≤ 0.05^48^. The traditional χ2 difference test was not used because it is typically used to compare the fit of two nested models and sensitive to sample size, thus tends to give significant results with moderate-to-large sample sizes^49^. Network estimation and resampling was performed with the *EGAnet* package(R Studio, version 4.1.0, Boston, MA, U.S.A.). To examine the practical value of new structure, we used generalized linear model (GLM) to examine the association between resilience dimensions from two structures with self-rated health(dichotomous variable, "excellent/good" vs."general/bad") as the dependent variable. The dimension scores of both structures were divided into quartiles to examine their associations with self-rated health.

## Results

### Demographic characteristics of the sample

The demographic distributions of the total and two separate sample are shown in Table 1. In the total sample, over a half were aged between 65 and 74 years old; 52.39% of the participants were female; over 80% of the participants were married. The distribution of education level was 35.82% for primary school and below, 33.46% for junior high school and 30.72% for senior high school and above, respectively. 41.04% participants rated their helath as "general”, 8.24% of older adults rated their health status as "bad”. We used Kruskal–Wallis test and Wilcoxon rank-sum test to compare demographic characteristics in both samples. Results indicated all demographic characteristics were not different between sample 1 and sample 2 (Table 1).

**Table 1:** Demographic characteristics distribution on the total and separate sample.

	*Total sample*(%)	*Sample 1*(%)	*Sample 2*(%)
Age (years)^1^			
65 ~	4257 (22.22)	2562 (22.29)	1695 (22.12)
70 ~	6234 (32.55)	3761 (32.72)	2473 (32.28)
75 ~	4507 (23.53)	2672 (23.25)	1835 (23.95)
80 ~	4157 (21.7)	2498 (21.73)	1659 (21.65)
Gender^2^			
Male	9120 (47.61)	5469 (47.59)	3651 (47.65)
Female	10,035 (52.39)	6024 (52.41)	4011 (52.35)
Education level^1^			
Primary school and below	6861 (35.82)	4173 (36.31)	2688 (35.08)
Junior high school	6409 (33.46)	3828 (33.31)	2581 (33.69)
Senior high school and above	5885 (30.72)	3492 (30.38)	2393 (31.23)
Marital status^2^			
Unmarried	3702 (19.33)	2227 (19.38)	1475 (19.25)
Married	15,453 (80.67)	9266 (80.62)	6187 (80.75)
Self-rated health^1^			
Excellent	2209 (11.53)	1355 (11.79)	854 (11.15)
Good	7506 (39.19)	4464 (38.84)	3042 (39.7)
General	7861 (41.04)	4733 (41.18)	3128 (40.82)
Bad&Very bad	1579 (8.24)	941 (8.19)	638 (8.33)

^1^Kruskal-Wallis test; ^2^Wilcoxon rank-sum test.

### Item analysis and standardized loading pattern matrix on total sample

For the total sample, we first adopted EFA (Kaiser–Meyer–Olkin (KMO): MSA = 0.98; Bartlett's sphericity test: *χ*^2^(300) = 431,501.5, *P* < 0.001) and parallel analysis suggested that the two factors structure was the model that best fit with the Chinese version of CD-RISC. After deleting any item in the scale, Cronbach’s α for the remaining items are all 0.97, which does not exceed 0.972. The mean inter-item-correlation was 0.591 and Cronbach’s α was 0.972 indicating items were internally consistent. Item discrimination criterion was set to 0.3 to check problematic items (poor retest-reliability or wording, semantic redundancy, differential item functioning, collinearity or rarity)^45,50^. Results showed all the 25 items were above the threshold of 0.3 as shown in Table 2.

**Table 2:** Item analysis on the total sample.

Item	Mean	SD	Skew	Item difficulty	Item discrimination	α if deleted
1	2.57	0.93	− 0.42	0.64	0.69	0.97
2	2.60	0.98	− 0.54	0.65	0.63	0.97
3	1.59	1.18	0.23	0.40	0.41	0.97
4	2.33	0.96	− 0.24	0.58	0.79	0.97
5	2.25	0.98	− 0.19	0.45	0.81	0.97
6	2.24	0.98	− 0.17	0.56	0.78	0.97
7	2.15	0.98	− 0.07	0.54	0.81	0.97
8	2.27	0.97	− 0.19	0.57	0.78	0.97
9	2.40	0.94	− 0.26	0.60	0.74	0.97
10	2.45	0.95	− 0.36	0.61	0.79	0.97
11	2.28	0.96	− 0.21	0.57	0.84	0.97
12	2.30	0.96	− 0.23	0.58	0.81	0.97
13	2.44	0.98	− 0.34	0.61	0.73	0.97
14	2.28	0.96	− 0.19	0.57	0.82	0.97
15	2.00	1.08	− 0.04	0.50	0.78	0.97
16	2.21	1.00	− 0.19	0.55	0.81	0.97
17	2.16	1.03	− 0.17	0.54	0.82	0.97
18	2.09	1.00	− 0.07	0.52	0.81	0.97
19	2.34	0.96	− 0.31	0.59	0.80	0.97
20	1.92	1.04	0.03	0.48	0.68	0.97
21	2.07	1.02	− 0.11	0.52	0.78	0.97
22	2.32	0.99	− 0.30	0.58	0.78	0.97
23	1.80	1.09	0.12	0.45	0.72	0.97
24	2.15	1.04	− 0.20	0.54	0.78	0.97
25	2.08	1.06	− 0.14	0.52	0.75	0.97

We then calculated the correlation coefficients matrix and remove the item with coefficients lower than 0.4. In Model 1, we firstly put all of the 25 items and the results showed that for item 3 (d503), for the loading for both factor 1 and 2 were lower than 0.4. The loading pattern matrix in Model 2 found that after removal of the item 3, all the left 24 items showed proper loadings on the two factors. Factor 1 explained 54% variance and Factor 2 accounted for 46%. The results are shown in Table 3.

**Table 3:** Standardized loading pattern matrix on the total sample.

	Model 1		Model 2	
	Factor 1	Factor 2	Factor 1	Factor 2
d501	*0.85*	−0.11	**0.85**	−0.11
d502	*0.82*	−0.15	**0.82**	−0.16
d503	0.11	0.33	–	–
d504	*0.73*	0.10	**0.74**	0.09
d505	*0.64*	0.23	**0.65**	0.22
d506	*0.65*	0.19	**0.65**	0.18
d507	*0.55*	0.31	**0.56**	0.30
d508	*0.70*	0.14	**0.70**	0.14
d509	*0.77*	0.02	**0.77**	0.02
d510	*0.82*	0.03	**0.81**	0.04
d511	*0.61*	0.28	**0.61**	0.28
d512	*0.64*	0.22	**0.64**	0.23
d513	*0.65*	0.13	**0.65**	0.14
d514	*0.55*	0.33	**0.55**	0.33
d515	0.19	*0.64*	0.20	**0.63**
d516	0.39	*0.48*	0.39	**0.48**
d517	0.18	*0.70*	0.18	**0.70**
d518	0.17	*0.70*	0.17	**0.70**
d519	*0.46*	0.38	**0.46**	0.39
d520	−0.03	*0.75*	−0.01	**0.85**
d521	−0.01	*0.85*	0.32	**0.52**
d522	0.32	*0.51*	−0.20	**0.97**
d523	−0.21	*0.98*	0.09	**0.75**
d524	0.09	*0.75*	0.07	**0.74**
d525	0.08	*0.74*	**0.85**	−0.11

Significant values are in bold and italic.

### EGA on sample 1 and sample 2

**Figure 1:**
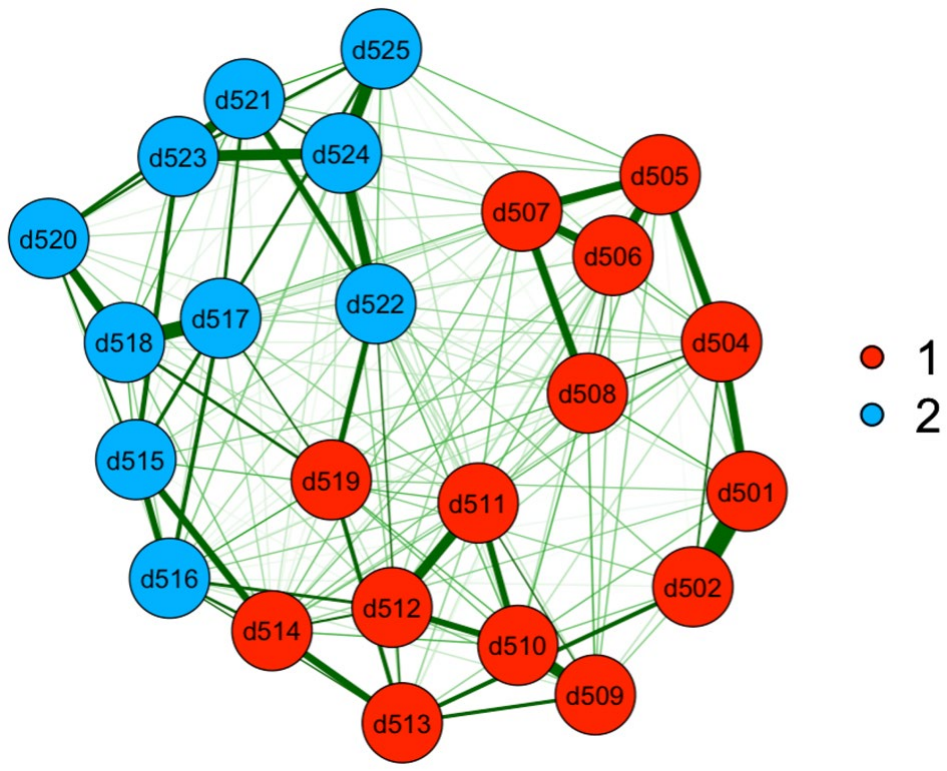
EGA results for sample 1.

**Figure 2:**
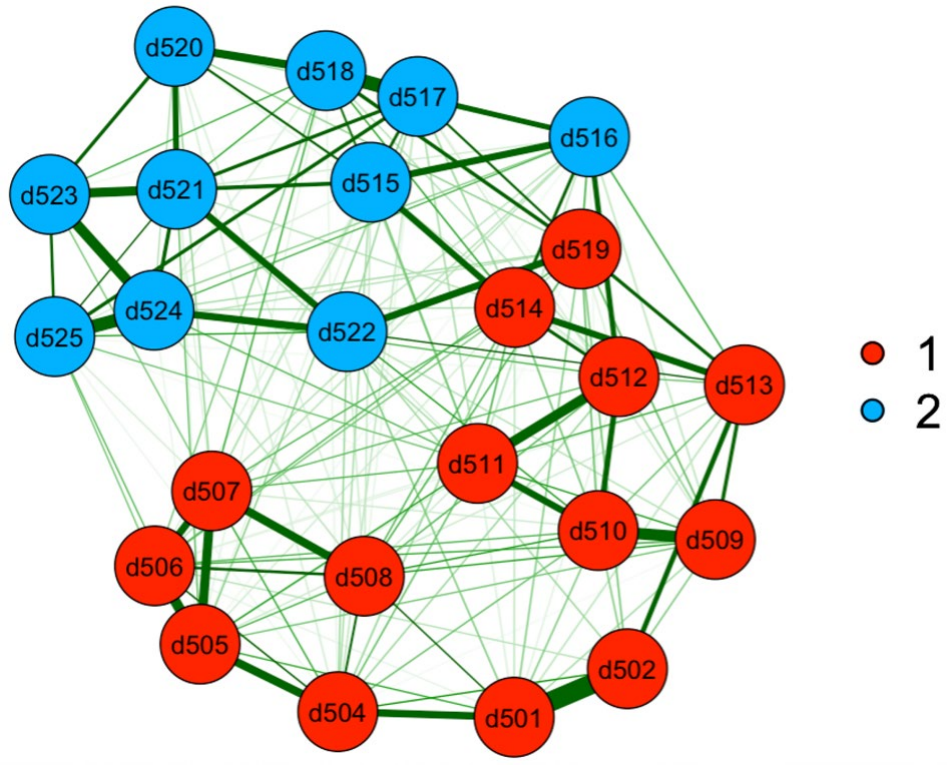
EGA results for sample 2.

**Figure 3:**
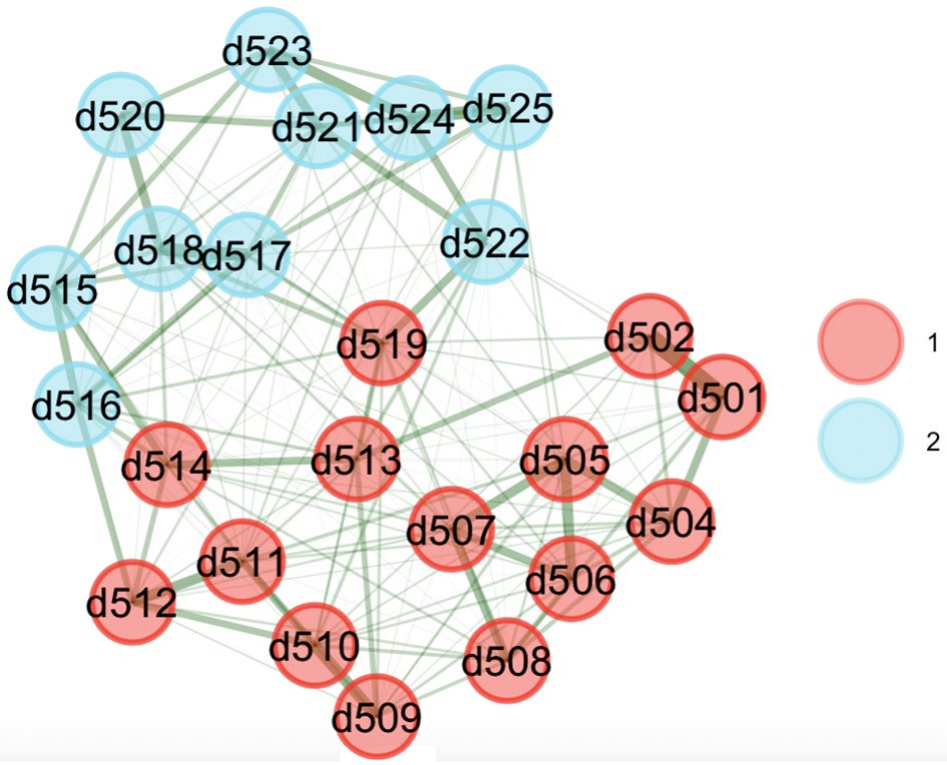
Bootstrapped EGA for sample 1.

**Figure 4:**
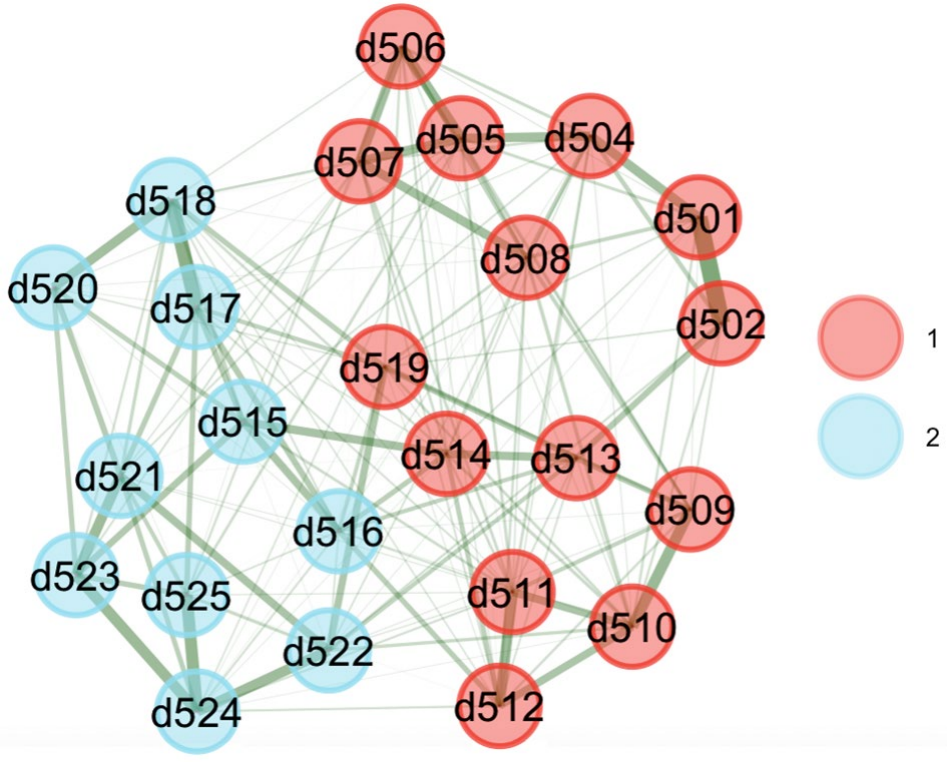
Bootstrapped EGA for sample 2.

EGA was conducted respectively among sample 1 and sample 2 to check the consistency. As shown in Figs. 1, 2, 3, 4, nodes of the networks represent the items, with item d501-d525 corresponds to item 1–25 of CD-RISC respectively (see Supplementary 1). Edges indicates the level of interaction between items, with higher value of correlation showing greater thickness.

The EGA analyses revealed two dimensions in the Chinese version of CD-RISC, we named as foresight and self-adjustment respectively. Notably, the item contents of 2 dimensions were identical in both samples. For the dimension of foresight, item 1, 2, 4–14, and 19 were largely connected to focus on reactions the individual adults imagine when encountering difficulties. The other dimension self-adjustment describes feelings of purpose and future orientation, consisting of item 15–18 and 20–25. The two dimensions are shown in different colors in Figs. 1 and 2.

We bootstrapped results over 1000 samples using the *bootEGA* function in *EGAnet* package. The network structural results showed good stability in both samples. Bootstrapped results are shown in Figs. 3 and 4.

### CFA comparison for EGA and original structures

After drawing the 2-factor structure from EGA, we compared the results with the original 3-factor structure by conducting CFA. As shown in Table 4, SRMR and TLI of both structures were ≤ 0.08 and > 0.90 respectively, with 2-factor structure performing better. CFI of 2-factor structure was above 0.95 while original 3-factor structure wasn’t. RMSEA of neither structures was ≤ 0.05(0.083 for original structure and 0.065 for 3-factor structure). Overall, the psychometric indices of EGA 2-factor structure performed better than the original 3-factor structure.

**Table 4:** CFA indices of 3-factor and 2-factor structure comparison.

	Chi-Squared	Degree of freedom	*P* value	CFI	TLI	SRMR	RMSEA	RMSEA90%CI
Original	13,436.931	251	< 0.001	0.923	0.915	0.037	0.083	(0.082,0.084)
EGA	8129.417	242	< 0.001	0.954	0.947	0.027	0.065	(0.064,0.066)

### GLM comparison for EGA and original structures

To assess the predictive value of the two dimensional structures, GLM was also conducted between the resilience scores and self-rated health. According to previous findings, GLM models controlled the following covariates: age, gender, marital status, educational level, as shown in Table 5 and Table 6. The results showed that compared to the original structure, the bi-factor found nearly all quartiles of both dimensions showed better predictive value of SRH. Compared with older adults with the lowest quartile, EGA results found a higher level of foresight predicted better SRH, with the second (odds ratio[OR] = 0.68, 95% confidence interval [95%CI] = 0.62 ~ 0.75), third (OR = 0.50, 95%CI = 0.45 ~ 0.56) and fourth quartile(OR = 0.42, 95%CI = 0.37 ~ 0.48). In the dimension of self-adjustment, the second (OR = 1.11, 95%CI = 1.01 ~ 1.23) and fourth (OR = 0.79, 95%CI = 0.69 ~ 0.90) quartile also showed homogeneous associations. In the original 3-factor structure, a higher level of strength predicted better SRH, with the second (OR = 0.77, 95% CI = 0.69 ~ 0.86), third (OR = 0.61, 95%CI = 0.54 ~ 0.69) and fourth quartile of strength (OR = 0.51, 95%CI = 0.43 ~ 0.59) showed a decreasing trend. There were few significant associations between optimism or tenacity with SRH.

**Table 5:** GLM results between original 3-factor structure and self-rated health.

	Odds ratio	std. err	z	*P*	95% CI
Cons	1.41	0.09	5.31	< 0.001	(1.24,1.61)
Strength					
1st quartile	1(Ref)				
2nd quartile	0.77	0.04	− 4.76	< 0.001	(0.69,0.86)
3rd quartile	0.61	0.04	− 7.57	< 0.001	(0.54,0.69)
4th quartile	0.51	0.04	− 8.45	< 0.001	(0.43,0.59)
Optimism					
1st quartile	1(Ref)				
2nd quartile	0.95	0.05	− 1.13	0.259	(0.86,1.04)
3rd quartile	0.86	0.05	− 2.81	0.005	(0.77,0.96)
4th quartile	0.84	0.05	− 2.83	0.005	(0.74,0.95)
Tenacity					
1st quartile	1(Ref)				
2nd quartile	0.99	0.05	− 0.26	0.796	(0.89,1.1)
3rd quartile	0.91	0.06	− 1.55	0.122	(0.8,1.03)
4th quartile	0.75	0.06	− 3.70	< 0.001	(0.65,0.87)
Age group					
65 ~	1(Ref)				
70 ~	1.25	0.05	5.48	< 0.001	(1.16,1.36)
75 ~	1.60	0.07	10.51	< 0.001	(1.46,1.75)
80 ~	1.71	0.08	11.31	< 0.001	(1.56,1.88)
Gender					
Male	1(Ref)				
Female	1.18	0.04	5.27	< 0.001	(1.11,1.26)
Education level					
Primary school and above	1(Ref)				
Junior high school	0.85	0.03	-4.49	< 0.001	(0.79,0.91)
Senior high school and above	1.03	0.04	0.64	0.52	(0.95,1.11)
Marital status					
Unmarried	1(Ref)				
Married	0.82	0.03	− 4.97	< 0.001	(0.76,0.89)

## Discussion

We adopted a novel method to explore the scale structure of Chinese version of CD-RISC in a large and representative sample and found that EGA produced a 2-factor structure (foresight and self-adjustment) that appeared preferably relevant for measuring the multidimensional nature of resilience and provided better data fit in indices of CFI, TLI, SRMR and RMSEA (95%CI). GLM results found that in the original structure, only strength and the 2 highest quantiles of optimism showed statistically significant value to predict SRH. In the dimension of tenacity, only the 4th quantile showed preventive effect on participants with lower SRH status. In the results of EGA structure, only the 3rd quantile of self-adjustment didn’t show predictive effect on older adults with poor SRH (as shown in Table 5 and Table 6).

**Table 6:** GLM results between EGA 2-factor structure and self-rated health.

	Odds ratio	std. err	z	*P*	95% CI
Cons	1.37	0.09	4.84	< 0.001	(1.20, 1.55)
Foresight					
1st quartile	1(Ref)				
2nd quartile	0.68	0.03	− 7.75	< 0.001	(0.62, 0.75)
3rd quartile	0.50	0.03	− 12.07	< 0.001	(0.45, 0.56)
4th quartile	0.42	0.03	− 12.88	< 0.001	(0.37,0.48)
Self-adjustment					
1st quartile	1(Ref)				
2nd quartile	1.11	0.05	2.19	0.028	(1.01,1.23)
3rd quartile	0.98	0.06	− 0.42	0.675	(0.87,1.09)
4th quartile	0.79	0.05	− 3.46	0.001	(0.69,0.90)
Age group					
65 ~	1(Ref)				
70 ~	1.25	0.05	5.48	< 0.001	(1.16,1.36)
75 ~	1.61	0.07	10.66	< 0.001	(1.47,1.76)
80 ~	1.71	0.08	11.3	< 0.001	(1.56,1.88)
Gender					
Male	1(Ref)				
Female	1.18	0.04	5.28	< 0.001	(1.11,1.26)
Education level					
Primary school and above	1(Ref)				
Junior high school	0.85	0.03	− 4.4	< 0.001	(0.79,0.91)
Senior high school and above	1.02	0.04	0.56	0.575	(0.95,1.10)
Marital status					
Unmarried	1(Ref)				
Married	0.82	0.03	− 4.98	< 0.001	(0.76,0.89)

In 2003, Connor and Davidson firstly developed a 5-factor scale named as Connor-Davidson Resilience Scale (CD-RISC-25)^37^. Then Burns and Anstey tested uni-dimensional structure of CD-RISC and further research has validated the unidimensional factor structure^51^. More recently, CD-RISC was validated in diverse cultural contexts such as South Africa^52^, Spain^53^, Canada^54^, Russia^55^, Greek^55^, ect. Validated studies of Chinese version of CD-RISC focused on specific groups under high-risk and high-pressure environments. Research found that in coal miners group, the resilience measurement consisted of two dimensions, namely tenacity and strength, with a total of 6 items^56^. In one study of parents with cancer-diagnosed children, Zeng etc. validated CD-RISC-10 and found single factor model was supported^57^. While the difference between these studies and ours may be explained from 2 aspects. Firstly, group differences should be noted. 6-item resilience scale focused on two dimensions, tenacity and strength, thus driving coal miners to achieve valuable or significant goals, at the same time enable coal miners to relax their emotions under difficult situations and avoid themselves from the influence of other pressure events, and thus be able to make decisions better. Scale structure in children patients underlied the association between resilience and distress, social support and found good psychometric properties of the unidimensional structure. To the best of our knowledge, none of the published research focused on CD-RISC in Chinese older population. We found that feedback regulation before (feedback forward, named as foresight) and after (feedback afterward, named as self-adjustment) distress may explain the resilience distribution in aging population. Additionally, previous validation in industry population and patient sample considered the number of items and volunteering bias, both of the above mentioned studies have reduced item numbers for making it much easier and less time consuming for respondents to answer the questions. Our study was based on a representative aging sample in China and the sample size may be efficient to genrelize resilience traits in Chinese older adults.

Herein we name the two dimensions as foresight and self-adjustment. As we found in EGA results, one dimension includes more items related closely with foresight. For example, item 1, 4, 6–12,14 and 19 depicted the foresight of an individual when encountering a difficult situation. According to Social Cognitive Theory^58^, human motivation and action are regulated by foresight, and this cognitive control of behavior is based on the self-efficacy expectations, which are the individuals’ beliefs in their capabilities to perform a course of action to attain a desired outcome. Several studies revealed that self-efficacy was a significant factor explaining resilience^59–62^, and resilience-building interventions targeted at older adults appeared effective in improving self-efficacy in at-risk older people^63^. The other 3 items in this dimension (2,5,13) described the buffing roles of resources. For example, item 2 "close and secure relationship" and item 13 "know where to turn for help" are related to social support, which has been discussed in other findings^64–66^, and item 5 "past success gives confidence for new challenge" concerned about the self confidence which may provide coping straregies. Zapater etc. found that under stressful condition, active coping strategies moderated a conditional indirect effect of resilience and cortisol reactivity among the older participants^67^. The other studies also implied that when facing the crisis of COVID-19, those older adults who better understood and promoted late-life coping (e.g. stay connected in physical isolation) were more resilient and less suffered from loneliness and sleep problems^68–70^. A review of resilience concept found older people who have the ability to use personal resources and see the world beyond their own concerns are more likely to be resilient^71^. The protective model of resilience was supported for goal orientation and self-confidence. Based on it, Askeland etc. found that goal orientation and self-confidence showed small moderating effects between negative life events and depressive symptoms^72^.

The other dimension self-adjustment includes item 15–18 and 20–25, which emphasized self-realization (item16, 17, 22, 23, 25) and the facet of future orientation (item 15, 18, 20, 21, 24). Self-adjustment generally equals to self-reinforcing, referring the process of reinforcing and maintaining one’s behavior with rewards that can be controlled when people achieve their own standards^73^. When people start doing something that may be difficult, their self-adjustment ability is affected by differences in stress ability, emotional response, and efforts to avoid negative behavior^74^. Herein we found the items in self-adjustment relates to self-realization and future orientation, which has been discussed elsewhere^75–77^. Self-realization and resilience were both aspects of well-being, found to correlate with disease self-management or difficulties among the older adults^78^. Studies elsewhere found self-realization was associated with quality of life positively and depression negatively^79^. As part of the psychological needs, when self-realization is not met, psychological state of the older people may change, which in turn affects their mental health^80^. As a self-initiated ability, future orientation (will to live) may help the development of resilience among maltreated youth^81^, children and older adults aged above 50 affected by HIV^82,83^ and the youth facing disasters^84^. A systematic review suggested that as transition to older age can challenge people's sense of self and their role in life^85^. One study foucused on older people in post-labor period of life found that older adults with higher self-realization and a moreoptimistic attitude to the future tend to enjoy beteer mental health status. As one study pointed that the resilience in older age was tied to an existential drive to create meaning in life and move toward a sense of self-fulfillment^86^, future interventions on how to help regain feelings of purpose and a fulfilling older age may help improve their resilience.

Finally, two limitations should be noted. First, we only used samples of older adults from China. Whether the findings can be generalized to samples from other countries should be further examined. Second, we only included measurement of subjective well-being as the outcome. Future studies should investigate whether the CD-RISC also has unique predictive effects on other well-being or health-related outcomes.

## Conclusion

In this study we conducted an in‐depth psychometric investigation of the Chinese version of CD-RISC using traditional (EFA and CFA) and contemporary (EGA) exploratory techniques. Although the scale was developed to assess three categories of resilience, our findings do not support the hypothesis that the scale delivers a psychometrically consistent measure of adult attachment styles. The results of the present study support the assumption that a two‐dimension approach (i.e., foresight and confidence) to assess resilience among older adults is optimal.

### Ethics approval and consent to participate

The Ethics Committee for Medical Research at a university approved the study (2019-04-0741). Written informed consent was obtained from all participants in the study. All methods were carried out in accordance with relevant guidelines and regulations.

### Supplementary Information

Supplementary Information.

## Data Availability

The datasets used and/or analysed during the current study are available from the corresponding author on reasonable request.
