# Supplementary material for: Exploratory graph analysis on the Connor–Davidson Resilience Scale (CD-RISC) among older adults in China
Source: Sci Rep. 2023 Nov 15;13:19971. doi: 10.1038/s41598-023-46854-x (PMC10651855; doi:10.1038/s41598-023-46854-x)
Supplement: Supplementary file 1 — Supplementary Information. [file 41598_2023_46854_MOESM1_ESM.pdf]

Supplementary 1 Item Summary with Dimensions of the CD-RISC-25

Item description	Dimension with description	Items by dimension
Able to adapt to change		
Close and secure relationships	optimism	2,3,4,6
Sometimes fate or God can help me		
Can deal with whatever comes		
Past success gives confidence for new challenge		
See the humorous side of things		
Coping with stress strengthens		
Tend to bounce back after illness or hardship		
Things happen for a reason		
Best effort no matter what	tenacity	11,12,13,14, 15,16, 17,18,19,20,21,22,23
You can achieve your goals		
Things look hopeless, I don't give up		
Know where to turn for help		
Under pressure, focus and think clearly		
Prefer to take lead in problem solving		
Not easily discouraged by failure		
Think of self as strong person		
Make unpopular or difficult decisions		
Can handle unpleasant feelings		
Have to act on a hunch		
Strong sense of purpose		
In control of your life		
I like challenges	strength	1,5,7.8,9,10,24,25
You work to attain your goals		
Pride in your achievements		

Data Availability Statement:
The datasets generated and/or analyzed during the current study are not publicly available due to necessity of interviewers' privacy but are available from the corresponding author on reasonable request.
